# Mechanism of virulence polymorphism in CR-hvKP strains from the same source

**DOI:** 10.1128/spectrum.02464-24

**Published:** 2025-05-23

**Authors:** Jianhua Fang, Hongyi Lai, Miao Deng, Yanfang Mei, Dehua Chen, Tieying Hou, Tianxin Xiang

**Affiliations:** 1 Jiangxi Provincial Key Laboratory of Prevention and Treatment of Infectious Diseases, The First Affiliated Hospital, Jiangxi Medical College, Nanchang University 74653, Nanchang, Jiangxi, China; 2 Infectious Diseases Department,The First Affiliated Hospital, Jiangxi Medical College, Nanchang University 74653, Nanchang, Jiangxi, China; 3 The First Clinical Medical College, Jiangxi Medical College, Nanchang University, Nanchang, Jiangxi, China; 4 Laboratory Department,The First Affiliated Hospital, Jiangxi Medical College, Nanchang University 74653, Nanchang, Jiangxi, China; 5 Medical Experimental Center, Shenzhen Nanshan People's Hospital, The Sixth Affiliated Hospital of Shenzhen University Medical School 481870, Shenzhen, Guangdong, China; Taichung Veterans General Hospital, Taichung, Taiwan

**Keywords:** virulent polymorphism, CR-hvKP, same source

## Abstract

Carbapenem-resistant *Klebsiella pneumoniae* (CRKP) has become a major public health issue worldwide due to its high case fatality rate and transmissible nature. The plasmid containing drug-resistant and virulence genes obtained by CRKP can evolve into carbapenem-resistant hypervirulent *Klebsiella pneumoniae* (CR-hvKP), thus forming a new generation of superbacteria. However, systematic documentation of the mechanism of plasmid evolution that carries both drug resistance and virulence genes remains lacking. Fifty-four strains of CR-hvKP were collected here. The clinical data and bioinformatics were analyzed to explore the genomics and virulence plasmid evolution mechanism from the same source in the strains PFGE1-ST11-K64, PFGE2-ST11-K64, PFGE-ST11-K2, and PFGE-ST11-K47. The four groups from the same source exhibited virulent polymorphism, and mutations in the *VirB11* gene may account for virulence variations across the strains of the same source.

**IMPORTANCE:**

This study emphasizes that in the process of clinical anti-infective treatment, attention should be paid not only to the strain itself but also to the external environment of the strain, especially the targeted therapeutic dose and course of host antibacterial drugs, which provides the possibility of diversity for the evolution of bacterial virulence and is conducive to the spread and survival of the CR-hvKP strain.

## INTRODUCTION

Carbapenem-resistant hypervirulent *Klebsiella pneumoniae* (CR-hvKP) is a hypervirulent, multidrug-resistant pathogen that has increasingly threatened public health in recent years due to its rapid transmission and high case fatality rate following infection (1). Moreover, carbapenem-resistant hypervirulent *Klebsiella pneumoniae* is responsible for both community-acquired and nosocomial infections, including pneumonia, liver abscess, urinary tract infection, and bloodstream infection, among others (2).

Carbapenem-resistant *Klebsiella pneumoniae* (CRKP) can evolve into CR-hvKP by the acquisition of mobile genetic elements that confer hypervirulence (3). The hypervirulent phenotype is mainly dependent on virulent genes and plasmids, such as *iuc* (the biosynthesis gene of ferriferic acidobacterin), *peg-344* (a metabolic transporter with unknown function), *rmpA*, and *rmpA2* (regulatory factors that increase capsule production), as demonstrated by relevant reports (4, 5). More importantly, drug resistance and virulence phenotype are consistent across CR-hvKP strains from the same source (6–8). However, the mechanisms underlying drug resistance and virulent polymorphism in the CR-hvKP strain from the same source are unclear. Thus, we investigate the mechanism of virulence polymorphism in CR-hvKP strains from the same source in this study through the analysis of clinical and bioinformatics data.

## MATERIALS AND METHODS

### Isolate identification

We collected bacterial isolates from sputum, blood, and other specimens. The strains were identified and tested for antimicrobial susceptibility using the French Vitek 2 system (bioMérieux). The susceptibility to imipenem and meropenem was determined using the disk diffusion method. The standardized protocol guidelines of the Clinical and Laboratory Standards Institute (M100-S32 document) were adopted.

Pulsed-field gel electrophoresis (PFGE), multilocus sequence typing (MLST), and K antigen typing (K) were employed to screen 54 strains of CR-hvKP according to the relevant literature (9).

### Serum resistance test

The serum resistance test was improved based on the literature method (10). Strains CRKP13, CRK41, ATCC25922, and NUHL30457 were resuscitated on Luria-Bertani (LB) solid medium, and appropriate strains were selected and diluted to 10^6^ CFU/mL. Serum from healthy subjects and inactivated serum were added to the diluted bacterial suspension for a certain period of time and coated with plates for overnight culture. Bacterial survival rates were calculated by counting the number of colonies on the two plates.

### Biofilm experiment

The biofilm experiment was modified with reference to relevant literature (11, 12). The bacteria were diluted to 0.5 McFarland turbidity, stained with crystal violet solution, dissolved in ethanol, and transferred to 96-well plates. The photochemical density (OD) of each well was determined. The negative control group (Nc) was determined by the mean ± triple the standard deviation ($\bar{X}$ ± 3 SD). The results were evaluated as follows: S strong positive (4 × Nc < OD); positive (2 × NC < OD ≤4 × Nc); weakly positive (Nc < OD ≤ 2 ×Nc); and negative (OD ≤ Nc).

### *Galleria mellonella* infection model

The *Galleria mellonella* infection model was used to preliminarily assess the virulence of the strain (13). The virulence of each strain was initially assessed using 10 *Galleria mellonella* weighing approximately 250 mg (purchased from Tianjin Huide Biotechnology, Tianjin, China). Each injection consisted of 10 µL at a concentration of 1 × 10^6^ CFU/mL. The survival of the moth was recorded every 12 hours for 3 days. All experiments were performed three times. NUHL30457 and ATCC700603 were used as controls for hypervirulence and hypovirulence strains, respectively.

### String test

The bacteria were incubated overnight on a sheep blood agar plate, and the single colonies were then wire-drawn with a vaccination ring. The string test was defined as positive when over 5 mm length of mucus was stretched (possibility of hypervirulence) (12, 13).

### PFGE, MLST, and K typing

The specific experimental scheme was operated with reference to relevant literature (12, 14). The clonal relationship and strain typing of CRKP isolates were evaluated by PFGE, MLST, and K typing. CR-hvKP isolates were divided into the following clones by PFGE cluster analysis, with 80% homology as the reference as described in https://bigsdb.pasteur.fr/klebsiella/, according to the seven housekeeping genes: *gapA*, *infB*, *MDH*, *pgi*, *phoE*, *rpoB*, and *tonB*. The capsule serotype-specific genes (*K1*, *K2*, *K5*, *K20*, *K54*, and *K57*) were amplified by PCR and sequenced as described previously.

### Whole-genome sequencing

The strains in this study were sequenced by Source Sequence Biotechnology Co., Ltd. (Shanghai), as follows. First, a 10KB SMRT Bell library was constructed, and then DNA adhesion enzymes, pure magnetic beads, and DNA fragments were screened. BluePipin fragments were connected, purified, and screened. The eligible DNA was divided into appropriate fragments using the Covaris g-TUBE. In addition, AMPure PB beads were used to screen and purify SMRT Bell libraries. The constructed library was measured with the Agilent 2100 Bioanalyzer to quantify the size of the insertions. HiSeq data were used on the PacBio platform for sequencing, assembly, and further proofreading.

### Comparative genomic analysis

A comparative genome study was conducted utilizing BLAST (https://blast.ncbi.nlm.nih.gov/) to identify two strains of *Klebsiella pneumoniae* genome sequences in relation to the reference strain ATCC25922, NUHL30457. A comparative genomic study of the three plasmids was conducted on the Mauve website utilizing the gene sequences of plasmid one from the CRKP13 and CRKP41 strains, as well as plasmid one from NUHL30457 . The VarScan website was utilized to obtain variation information for the two strains from high-throughput sequencing data, accompanied by a comprehensive analysis of the single-nucleotide polymorphism (SNP) results. The genetic disparities between the two strains were compared. The *VirB11* gene sequences of KP13 and KP41 were analyzed to identify variations in the *VirB11* gene between the two strains.

### Real-time quantitative PCR for transcription analysis

RNA extraction and transcription were performed as previously described (15). Real-time quantitative PCR was used to measure the expression of *VirB11* and *16S rRNA* genes using the primers described earlier. The ΔΔCT method was used to normalize the *16SrRNA* gene with those of two strains from the same origin. The 16S rRNA gene was used as the internal control.

### Proteomic analysis of CRKP13 and CRKP41

Protein extraction, peptide analysis, and liquid chromatography-tandem mass spectrometry (LC-MS/MS) using data collection and database retrieval were performed for the mass spectrometry process (16). RIPA lysate and protease K were used to extract bacterial protein. The C18 membrane-filled column was used for desalting. The Nano-HPLC liquid phase system Easy 1200 was used for mass spectrometry and chromatography. Data analysis and identification were performed through the uniprot_*Klebsiella_pneumoniae*_subsp.pneumoniae_UP000007841_1125630 database.

### *VirB11* gene mutations of the CRISPR/Cas9 gene editing technique

In the study, the *VirB11* gene in the KP13 strain mutates C to G at position 712, and the mutant strain is named KP13V. In this study, the CRISPR/Cas9-mediated genome editing method was used for single base mutation of genes. PCR was used to amplify homologous sequences and vectors of target genes. The target DNA fragments were recovered, and the cloned products were seamlessly cloned and transformed into KP13 electroreceptive cells, coated on Hyg (100 µg/mL) LB plate, and cultured overnight. After screening positive transformants by PCR and Sanger sequencing, monoclonal colonies were selected and cultured at 37°C. The KP13-targeting plasmid (pCasPA-hyg-up-dn-sgRNA1-Kp13) was purified, and Sanger sequencing verified the intended point mutation.

### Method to study the effect of the *VirB11* gene mutation on the virulence and growth ability of the KP13 strain

The method of virulence test was the same as the above method of serum resistance, macroborer, and biofilm test, and the method of growth curve was referred to the relevant literature (14). Strains KP13 and KP13V were diluted to appropriate concentrations, and each culture (200 µL) was repeated three times into a flat-bottom 100-well plate. The plate was stirred and incubated at 37 ℃ for 24 h, and the automatic microbial growth curve analysis system was adopted by Bioscreen C.

## RESULTS

### Clinical characteristics of 11 patients infected with CR-hvKP isolates from the same source

We divided 54 strains of CR-hvKP into four groups, and the general characteristics are shown in Table 1. PFGE, MLST, and K types (PFGE1-ST11-K64, PFGE2-ST11-K64, PFGE-ST11-K2, and PFGE-ST11-K47) all showed that the strains were from the same source. Three strains of PFGE1-ST11-K64, 2 strains of PFGE2-ST11-K64, 4 strains of PFGE-ST11-K2, and 2 strains of PFGE-ST11-K47 were identified, resulting in a total of 11 strains. The clinical characteristics of patients infected with these 11 CR-hvKP isolates from the same source summarization are shown in Table 2. The average age of infected patients among the four groups of strains from the same source was 60 years old, with a predominance of elderly populations. Of these infected patients, 81.8% underwent an invasive procedure without requiring transfer to another center. Of these patients, 72.7% had a history of intensive care unit admission; the average length of hospital stay was 27 days; and about 63.6% of the patients had a poor prognosis, including death and deterioration.

**TABLE 1:** General characterization of 54 CR-hvKP strains^*a*^

Strain	Source	K type	ST	String test	Carbapenemase	Virulence gene												
						*prmpA*	*prmpA2*	*terW*	*kpu*	*iutA*	*mrkD*	*entB*	*fimH*	*Sils*	*wcaG*	aerobactin	*magA*	*peg344*
KP1	Blood	K2	11	−	KPC	1	1	1	0	1	0	1	0	0	0	0	0	0
KP2	Blood	K1	11	+	0	1	1	1	0	1	0	1	0	0	0	0	0	1
KP3	Blood	K47	11	−	KPC	1	1	1	0	1	0	1	0	0	0	0	0	1
KP4	Blood	K2	11	−	KPC	1	1	1	0	1	0	1	0	0	0	0	0	1
KP5	Other	K2	11	−	KPC	1	1	1	0	1	0	1	0	0	0	0	0	0
KP6	Blood	K1	11	−	KPC	1	1	1	0	1	0	1	0	0	0	0	0	0
KP7	Blood	K2	11	−	KPC	1	1	1	0	1	0	1	0	0	0	0	0	0
KP8	Blood	K2	11	−	KPC	1	1	1	0	1	0	1	0	0	0	0	0	0
KP9	CSF	K64	11	−	0	1	1	1	0	1	0	1	0	0	0	0	0	0
KP10	Blood	K2	11	−	KPC	1	1	1	0	1	0	1	0	0	0	0	0	0
KP11	Blood	unknown	11	−	KPC	1	1	1	0	1	0	1	0	0	0	0	0	0
KP12	Blood	K2	11	−	KPC	1	1	1	0	1	0	1	0	0	0	0	0	0
KP13	Blood	K2	11	+	0	1	1	1	0	1	0	1	0	0	0	0	0	0
KP14	CSF	K64	11	−	KPC	1	1	1	0	1	0	1	0	0	0	0	0	0
KP15	Blood	K1	23	+	0	1	1	1	0	1	0	1	0	0	0	1	0	1
KP16	Blood	K47	11	−	KPC	1	1	1	0	1	0	1	0	0	0	0	0	0
KP17	Blood	K64	11	−	KPC	1	1	1	0	1	0	1	0	0	0	0	0	0
KP18	Other	K47	11	−	KPC	1	1	1	0	1	0	1	0	0	1	1	0	1
KP19	Blood	K47	163	+	0	1	1	1	0	1	0	1	0	0	0	1	0	1
KP20	Blood	unknown	515	−	KPC	1	1	1	1	1	1	1	1	1	0	0	1	1
KP21	Blood	K1	3492	+	0	1	1	1	1	1	1	1	1	1	0	1	1	1
KP22	Blood	K2	11	+	KPC	1	1	1	1	1	1	1	1	1	0	0	0	0
KP23	Blood	K1	3055	−	KPC	1	1	1	1	1	1	1	1	1	0	1	1	1
KP24	Blood	K2	65	+	KPC	1	1	1	1	1	1	1	1	1	0	1	0	1
KP25	Blood	K2	11	−	KPC	1	1	1	1	1	1	1	1	1	1	1	0	1
KP26	Blood	K1	23	+	0	1	1	0	1	1	1	1	1	0	1	1	0	1
KP27	Blood	K1	23	+	0	1	1	1	1	1	1	1	1	0	1	1	0	0
KP28	Blood	K1	23	−	KPC	1	1	1	1	1	1	1	1	1	1	1	0	1
KP29	Blood	K2	11	+	KPC	1	1	1	1	1	1	1	1	1	1	1	0	1
KP30	Blood	K1	1265	+	0	1	1	1	1	1	0	1	1	1	1	1	0	1
KP31	Blood	unknown	519	+	0	1	1	1	1	0	0	1	1	1	0	1	0	1
KP32	Blood	unknown	11	−	KPC	1	1	1	1	1	0	0	1	1	1	0	0	1
KP33	Blood	K2	11	−	KPC	1	1	1	0	1	0	1	0	0	1	0	0	0
KP34	Blood	K47	11	+	KPC	1	1	1	0	1	0	1	0	0	1	0	0	0
KP35	Blood	K1	11	+	KPC	1	1	1	1	1	1	1	1	1	1	1	1	1
KP36	Blood	K64	11	−	KPC	1	1	1	0	1	0	1	0	0	1	0	0	0
KP37	Blood	K64	11	−	KPC	1	1	1	0	1	0	1	0	0	1	1	0	1
KP38	Blood	K1	11	−	KPC	1	1	1	0	1	0	1	0	0	1	0	0	0
KP39	Blood	K64	11	+	KPC	1	1	1	0	1	0	1	0	0	1	1	0	1
KP40	Blood	K47	11	+	KPC	1	1	1	1	1	1	1	1	1	1	1	0	1
KP41	Blood	K2	11	−	KPC	1	1	1	1	1	1	1	1	1	1	0	0	1
KP42	Blood	K47	11	−	KPC	1	1	1	1	1	1	1	1	1	0	0	0	1
KP43	Blood	unknown	11	−	KPC	1	1	1	1	1	1	1	1	1	0	0	0	0
KP44	Blood	K64	11	−	KPC	1	1	0	1	1	1	1	1	1	0	0	0	0
KP45	Blood	K64	11	−	KPC	1	1	1	0	1	0	1	0	0	0	0	0	1
KP46	Blood	K47	11	−	KPC	1	1	1	0	1	0	1	0	0	0	0	0	0
KP47	Blood	K64	11	−	KPC	1	1	1	0	1	0	1	0	0	0	0	0	1
KP48	Other	K47	11	−	KPC	1	1	1	1	1	1	1	1	1	0	0	0	0
KP49	Other	K2	524	−	KPC	1	1	1	1	1	1	1	1	1	0	0	0	0
KP50	Blood	K2	5	−	KPC	1	1	1	0	0	0	1	0	0	0	0	0	0
KP51	Blood	K64	11	−	KPC	1	1	1	1	1	1	1	1	1	0	0	0	0
KP52	CSF	K64	11	−	KPC	1	1	1	1	1	1	1	1	1	0	0	0	0
KP53	CSF	K64	11	−	KPC	1	1	1	1	1	1	1	1	1	0	0	0	0
KP54	Blood	K2	11	−	KPC	1	1	1	1	1	1	1	1	1	1	1	0	0

^*a*^ CSF, cerebrospinal fluid; 1 represents presence, and 0 represents absence; − represents negative, and + represents positive.

**TABLE 2:** Clinical data of strains from the same source (PFGE, ST, and K classification)

Strain	Department	Gender	Age (years)	Sample source	Prognosis	Invasive procedures	Long-term steroid use	Antibiotic usage	ICU admission	Department transfer	Length of hospital stay (days)
KP14	Respiratory ICU	Male	72	Blood	Improved	Yes	Yes	Meropenem, ertapenem, cefminox, polymyxin, and cefotaxime	Yes	Yes	10
KP45	ICU	Male	28	Blood	Dead	Yes	Yes	Tigecycline, imipenem, polymyxin, and micafungin	Yes	Yes	24
KP47	ICU	Male	56	Blood	Dead	Yes	Yes	Tigecycline, imipenem, polymyxin, fosfomycin, and piperacillin-tazobactam	Yes	Yes	67
KP13	ICU	Male	65	Blood	Improved	Yes	Yes	Meropenem, linezolid, tigecycline, micafungin, and piperacillin-tazobactam	Yes	No	59
KP29	Respiratory ICU	Female	68	Ascitic fluid and pleural effusion	Dead	Yes	Yes	Meropenem, micafungin sodium, polymyxin, and tigecycline	Yes	No	20
KP33	ICU	Female	42	Blood	Dead	Yes	Yes	Meropenem, ertapenem, polymyxin, cefminox, polymyxin, and cefotaxime	Yes	No	20
KP41	Hematology	Male	76	Blood	Worsen	Yes	Yes	Ceftobiprole, Linezolid, Teicoplanin, Tigecycline, Caspofungin,Voriconazole	No	No	42
KP46	ICU	Female	86	Blood	Dead	Yes	No	Biapenem	No	No	5
KP48	Neurology	Male	80	Blood	Improved	No	No	Ceftobiprole	No	No	5
KP51	Respiratory ICU	Male	59	Blood	Improved	Yes	No	Meropenem, tigecycline, linezolid, tigecycline, micafungin, and piperacillin-tazobactam	Yes	No	33
KP53	ICU	Female	35	Blood	Dead	Yes	No	Meropenem, polymyxin, and daptomycin	Yes	No	9

### Virulence-related gene, carbapenemase gene, capsule serotype gene, and MLST

The distribution results of carbapenemase genes, virulence-related genes, capsular serotype genes, and MLST in 11 CR-hvKP isolates are shown in Table 3. MLST analysis showed that there were a total of six sequence types (STs), with ST11 dominating the STs (45.5%, 5 of 11), and the rest (ST23, ST690, ST833, and ST5365) accounted for 9.1% (1 of 16). K2 was the most common serotype (*n* = 4), followed by K1 (*n* = 1), K64 (*n* = 3), and K47 (*n* = 1). The positive results of 13 virulence genes (*entB*, *fimH*, *rmpA2*, *iutA*, *iucA*, etc.) in 11 CR-hvKP isolates are shown in Table 2. The carbapenemase gene was mainly *KPC*, accounting for 90.9%.

**TABLE 3:** General characteristics of the bacterial strains in this study^*a*^

Strain	Sample source	ST	K type	String test	Carbapenemase	Virulence gene												
						*prmpA*	*prmpA2*	*terW*	*Kpu*	*iutA*	*mrkD*	*entB*	*fimH*	*Sils*	*wcaG*	aerobactin	*magA*	*peg344*
KP13	Blood	11	K2	+	0	1	1	1	0	1	0	1	0	0	0	0	0	0
KP14	CSF	11	K64	−	KPC	1	1	1	0	1	0	1	0	0	0	0	0	0
KP29	Blood	11	K2	+	KPC	1	1	1	1	1	1	1	1	1	1	1	0	1
KP33	Blood	11	K2	−	KPC	1	1	1	0	1	0	1	0	0	1	0	0	0
KP41	Blood	11	K2	−	KPC	1	1	1	1	1	1	1	1	1	1	0	0	1
KP45	Blood	11	K64	−	KPC	1	1	1	0	1	0	1	0	0	0	0	0	1
KP46	Blood	11	K47	−	KPC	1	1	1	0	1	0	1	0	0	0	0	0	0
KP47	Blood	11	K64	−	KPC	1	1	1	0	1	0	1	0	0	0	0	0	1
KP48	Other	11	K47	−	KPC	1	1	1	1	1	1	1	1	1	0	0	0	0
KP51	Blood	11	K64	−	KPC	1	1	1	1	1	1	1	1	1	0	0	0	0
KP53	CSF	11	K64	−	KPC	1	1	1	1	1	1	1	1	1	0	0	0	0

^*a*^ CSF, cerebrospinal fluid; 1 represents presence, and 0 represents absence; − represents negative, and + represents positive.

### Virulence polymorphisms of strains of the same origin (PFGE, ST, and K)

We split 54 strains of CR-hvKP into four types (PFGE1-ST11-K64, PFGE2-ST11-K64, PFGE-ST11-K2, and PFGE-ST11-K47) based on the same type of PFGE, MLST, and K. The serum resistance experiment, biofilm experiment, infection experiment, and high mucous phenotype experiment showed polymorphism among these four strains from the same source.

#### Virulence polymorphism of the PFGE1-ST11-K64 strain

**Fig 1:**
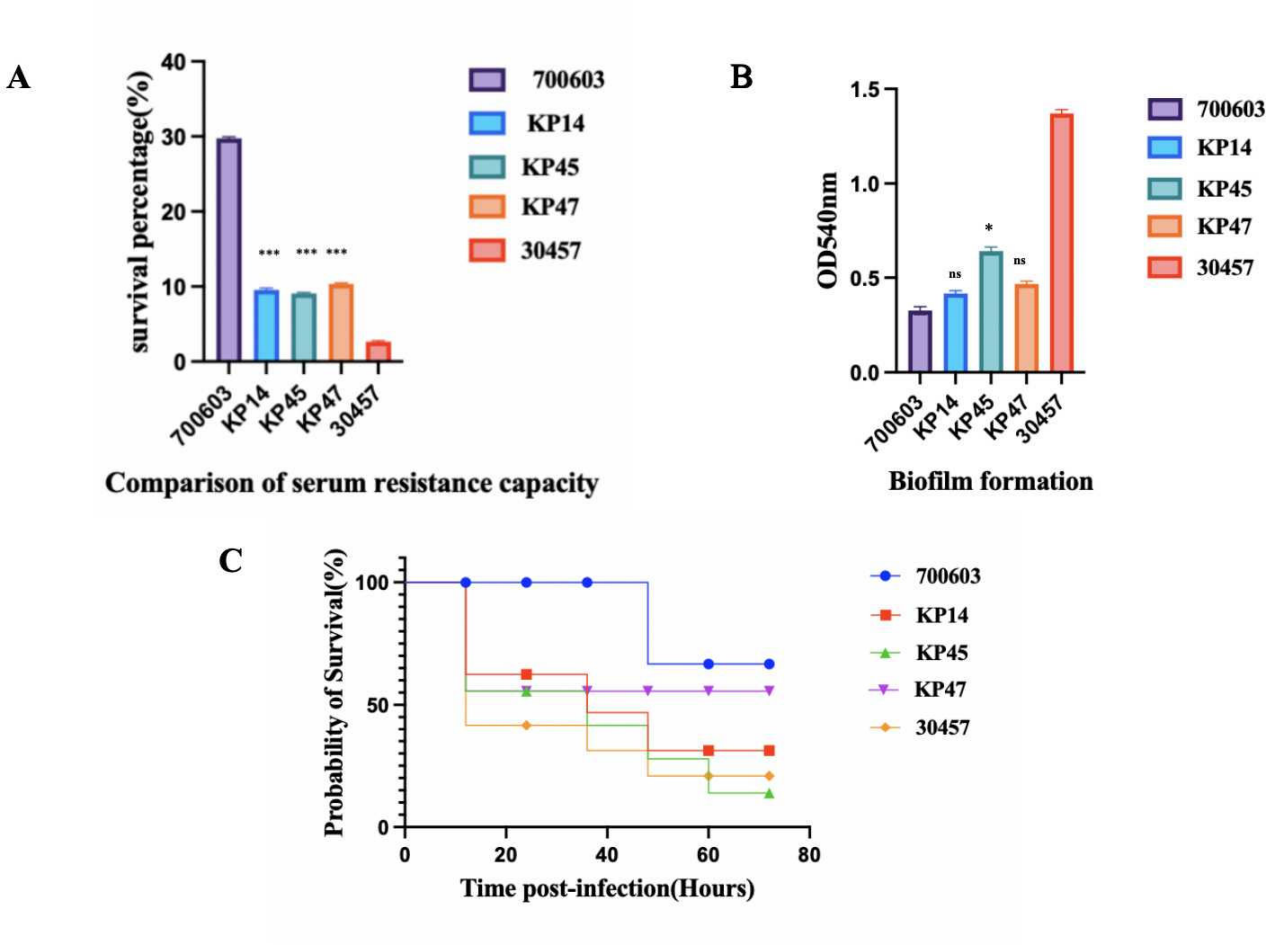
Virulent polymorphism of the PFGE1-ST11-K64 strain. (**A**) Serum resistance capability of KP14, KP45, and KP47. The serum resistance of KP47 is higher than that of KP14 and KP45. (**B**) The biofilm-forming ability of KP45 is higher than that of KP14 and KP47. (**C**) Virulence analysis of strains KP14, KP45, and KP47 in the *Galleria mellonella* infection model. *P < 0.1, ***P < 0.001. ns, no significance.

The three strains from the same source did not have the same level of virulence in the experiments of serum resistance, biofilm, and *Galleria mellonella* (Fig. 1).

#### Virulence polymorphism of the PFGE-ST11-K2 strain

**Fig 2:**
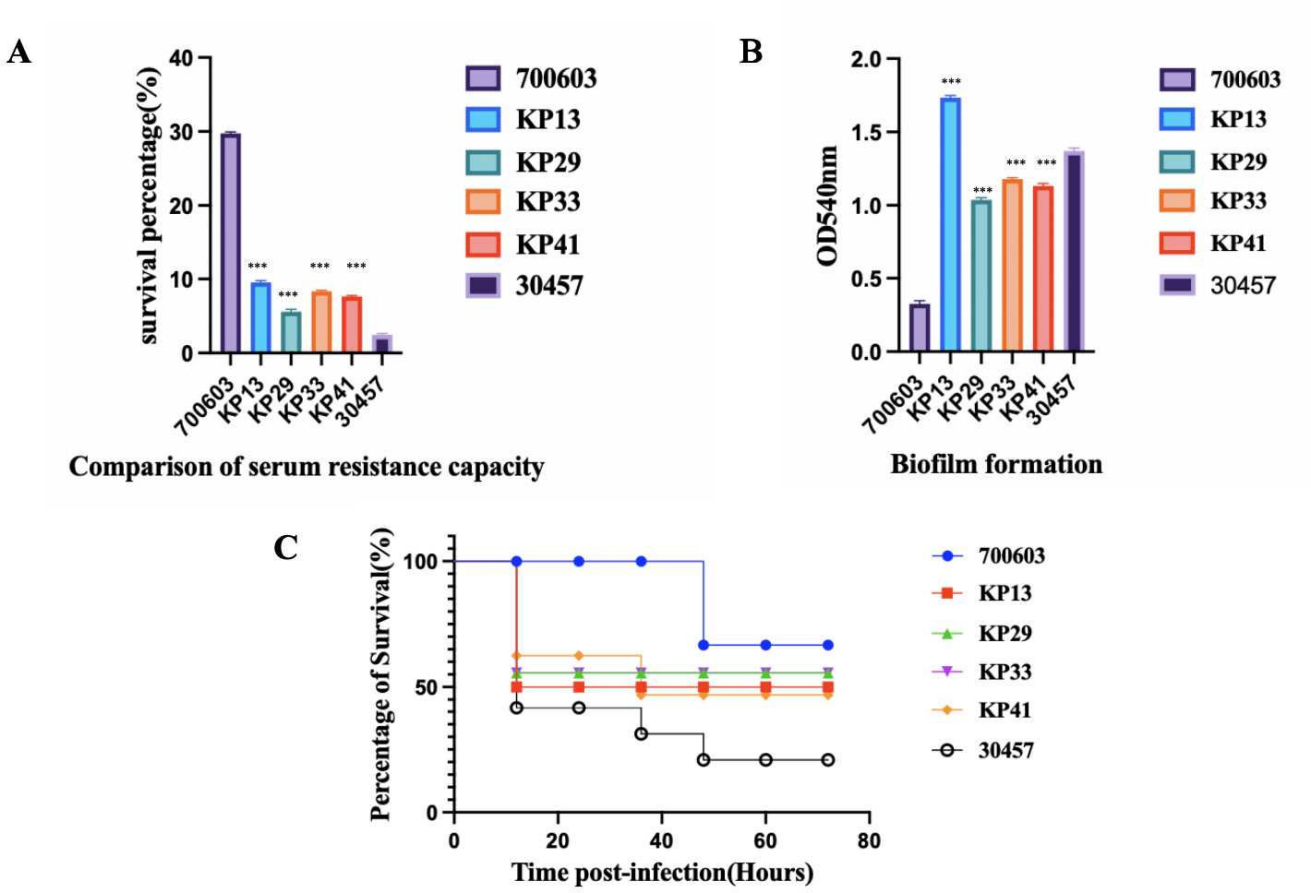
Virulent polymorphism of the PFGE-ST11-K2 strain. (**A**) Serum resistance capability of KP13, KP29, KP33, and KP41. The serum resistance of KP13 is higher than that of KP29, KP33, and KP41. (**B**) The biofilm-forming ability of KP13 is higher than that of KP29, KP33, and KP41. (**C**) Virulence analysis of strains KP13, KP29, KP33, and KP41 in the *Galleria mellonella* infection model. ***P < 0.001.

There was little difference in virulence between the four strains of the same source in the infection test of *Galleria mellonella* and biofilm formation, but in the serum resistance test, the virulence of KP33 and KP41 was significantly different from that of KP13 and KP29, as shown in Fig. 2.

#### Virulence polymorphism of the PFGE-ST11-K47 strain

**Fig 3:**
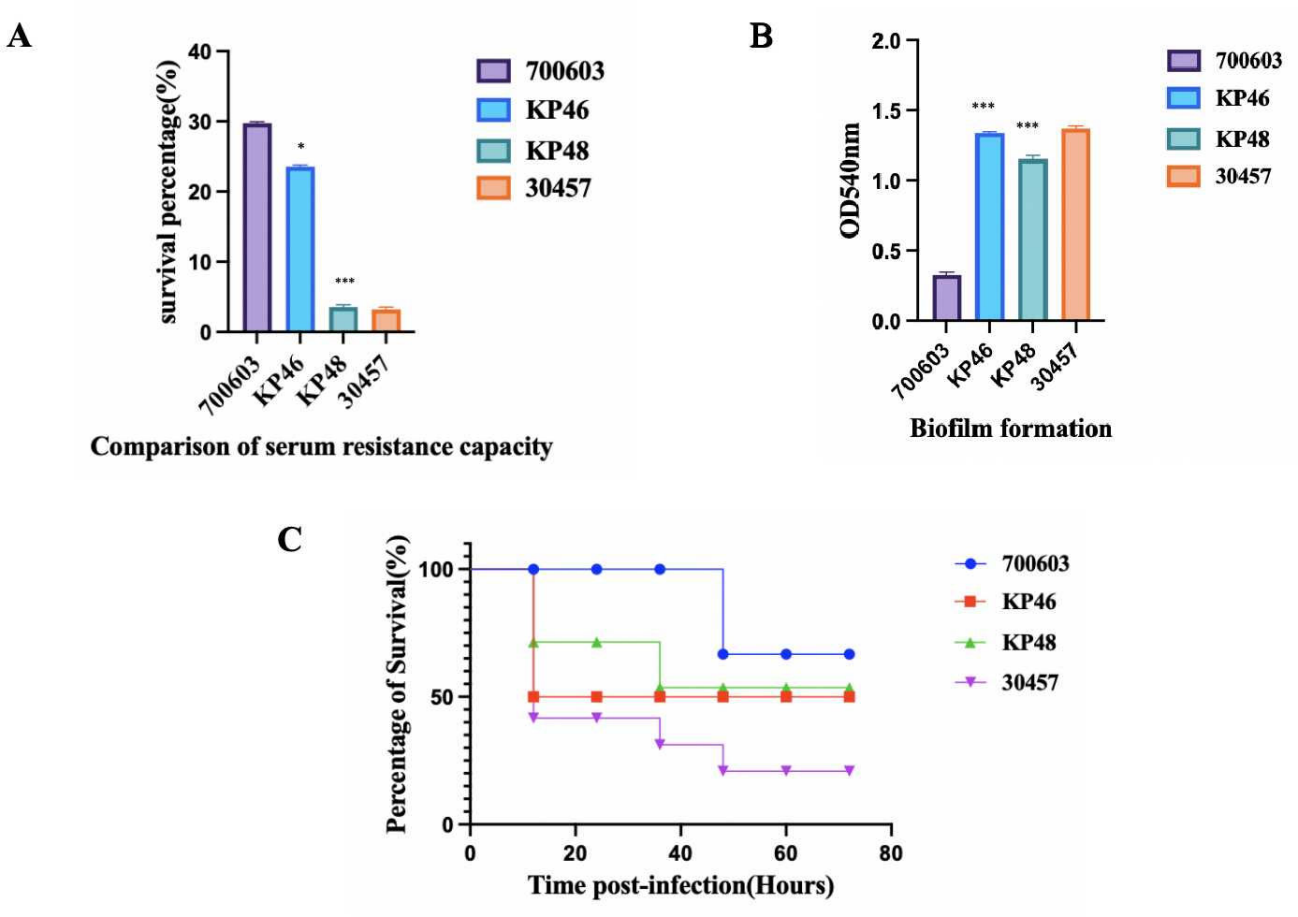
Virulent analysis of the PFGE-ST11-K47 strain. (**A**) Serum resistance capability of KP46 and KP48. The serum resistance of KP46 is higher than that of KP48. (**B**) The biofilm-forming ability of KP13 is higher than that of KP46 and KP48. (**C**) Virulence analysis of strains KP46 and KP48 in the *Galleria mellonella* infection model. *P < 0.1, ***P < 0.001.

There was no significant difference in virulence between KP46 and KP48 strains from the same source with regard to serum resistance, biofilm formation, and infection of *Galleria mellonella*, as shown in Fig. 3.

#### Virulence polymorphism of the PFGE2-ST11-K64 strain

**Fig 4:**
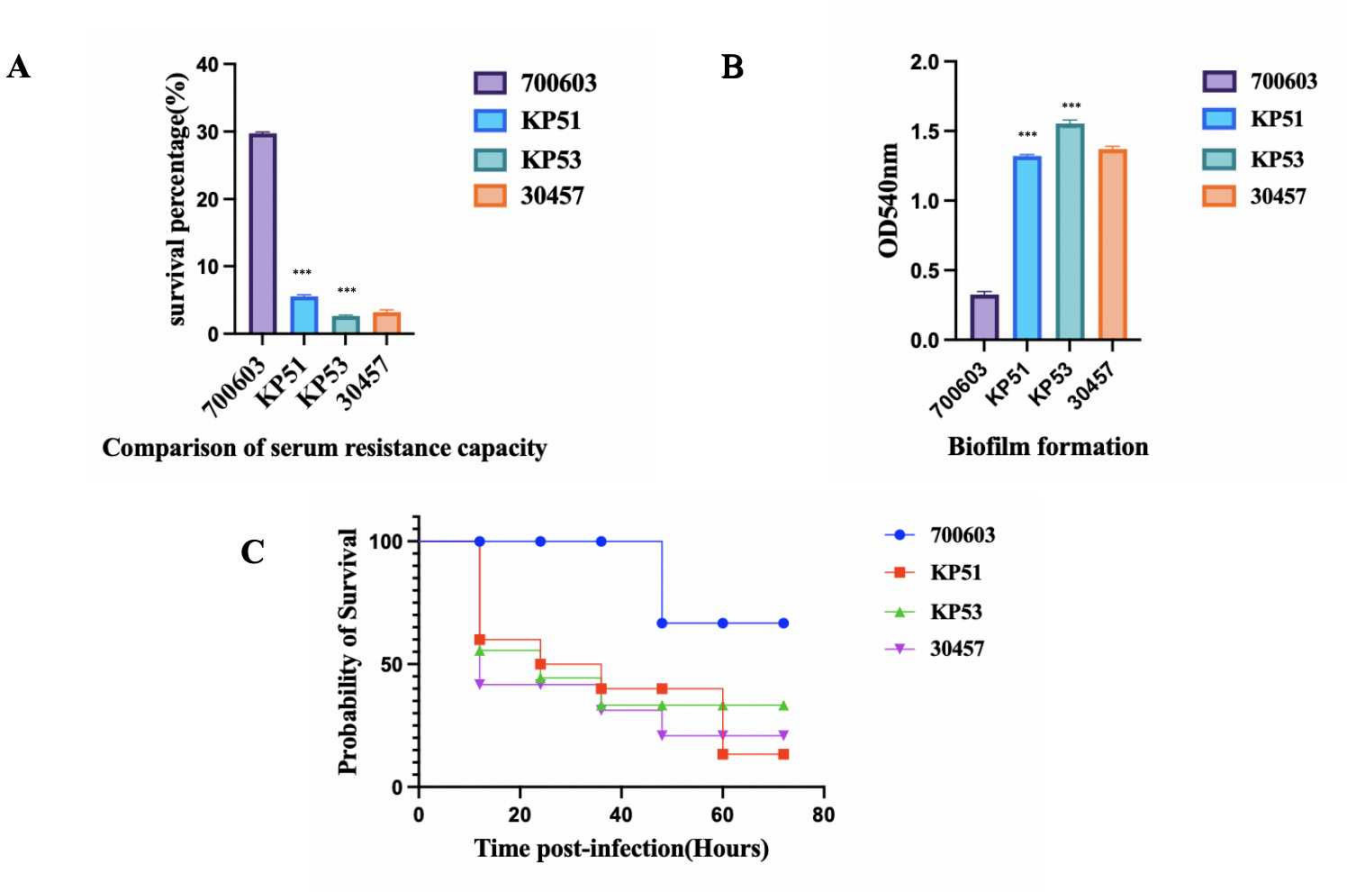
Virulent polymorphism of PFGE2-ST11-K64. (**A**) Serum resistance capability of KP51 and KP53. The serum resistance of KP51 is higher than that of KP53. (**B**) The biofilm-forming ability of KP53 is higher than that of KP51. (**C**) Virulence analysis of strains KP51 and KP53 in the *Galleria mellonella* infection model. ***P < 0.001.

The virulence of KP51 and KP53 exhibited minimal variation in the serum resistance and biofilm formation experiments for the two strains from the same source; however, KP53 demonstrated marginally greater virulence than KP51. Conversely, in the *Galleria mellonella* infection experiment, KP51 displayed superior virulence compared to KP53, as shown in Fig. 4.

### Genomics and virulence evolution mechanism of the same PFGE-ST11-K2 strain

Whole-genome analysis showed that KP13 contained one chromosome with 5,504,427 bp and four plasmids with sizes of 195,854, 139,321, 87,097, and 23,943 bp, respectively. The GC content was 56.9%. KP41 also carried one chromosome and four plasmids, the sizes of which were 5,459,370, 195,838, 144,993, 87,095, and 11,970 bp, respectively, and the GC content was 57%, as shown in Table 4.

**TABLE 4:** General characteristics of KP13 and KP41 genomes of *Klebsiella pneumoniae*

Sample	Chr1 length (bp)	p1 length (bp)	p2 length (bp)	p3 length (bp)	p4 length (bp)	Total length (bp)	GC content (%)
KP13	5,504,427	195,854	139,321	87,097	23,943	5,950,642	56.9
KP41	5,459,370	195,838	144,993	87,095	11,970	5,899,266	57.0

**Fig 5:**
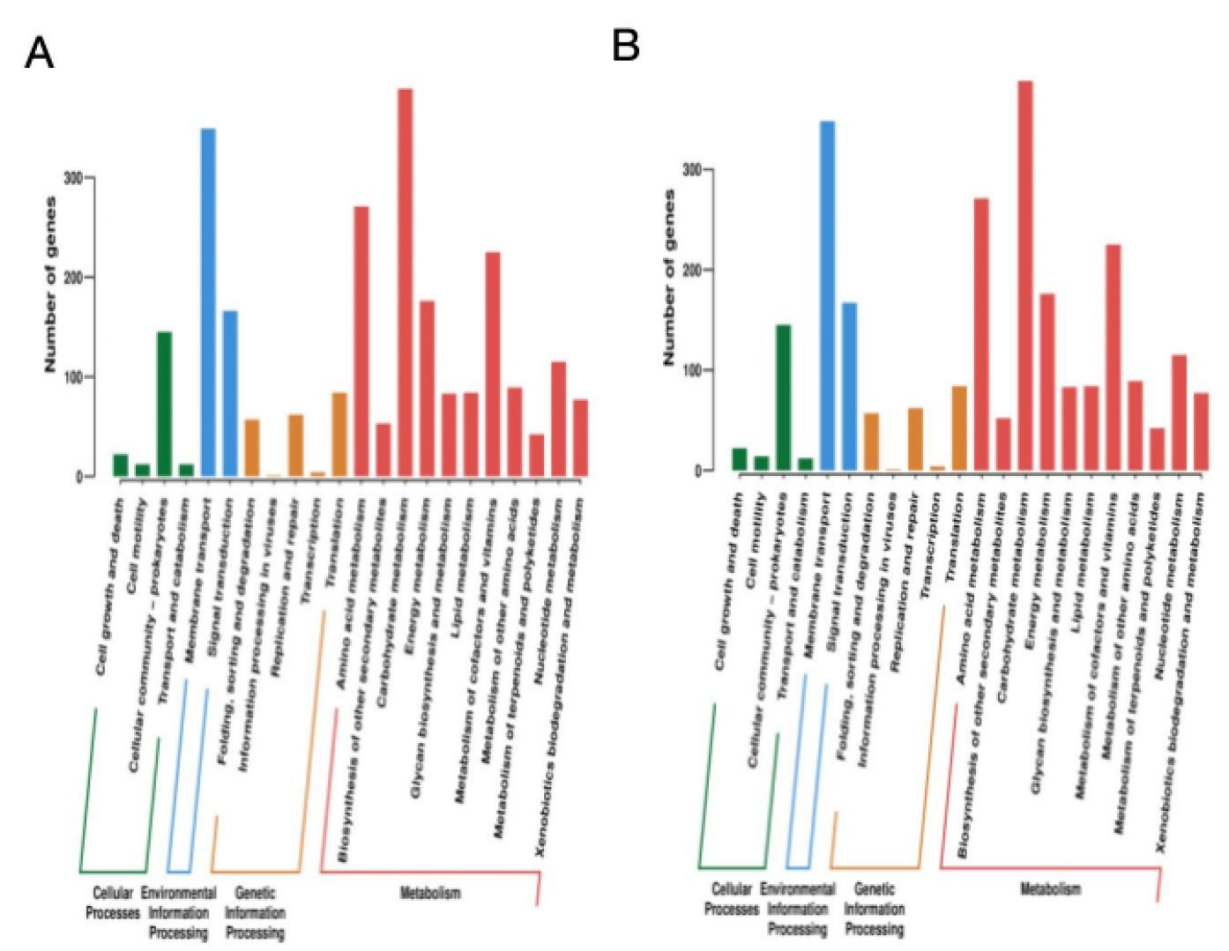
Comparison of gene function. (**A**) KEGG function annotation of strain KP13. (**B**) KEGG functional annotation of strain KP41.

Kyoto Encyclopedia of Genes and Genomes (KEGG) annotation was performed on 2,518 genes of KP13 and KP41 strains (Fig. 5). The results showed that most of the annotated genes of KP41 strains were related to basic life activities, among which carbohydrate metabolism was the most common. A total of 349 genes were associated with transmembrane transport. A total of 271 genes were related to amino acid metabolism, and the others were closely related to bacterial signal transduction, bacterial growth and death, transport, and catabolism.

### Comparative genomic analysis

**Fig 6:**
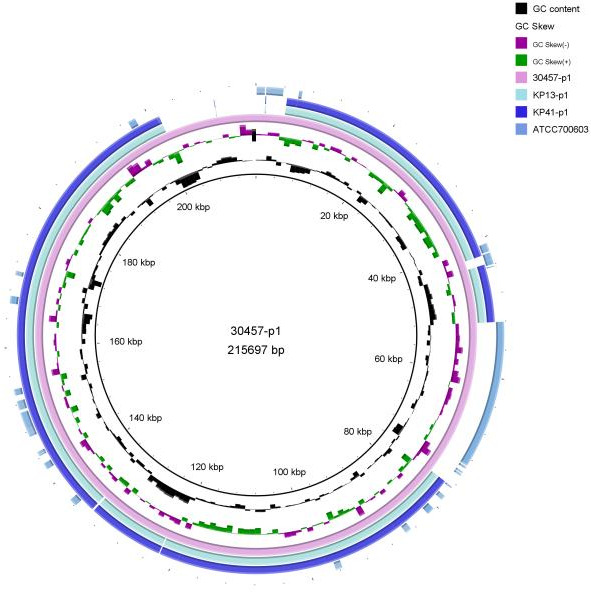
Comparison of the genomic circle map of KP13 and KP41. Each circle contains multiple virulence and resistance genes. The innermost circle is the positional coordinates of the genomic sequence. The gene circle diagrams for the 30457-p1 plasmid, KP13-p1 plasmid, and KP41-p1 plasmid are shown in order from inner to outer.

There was minimal genetic variation between strains KP13 and KP41 in terms of virulence and drug resistance. The *bla*_KPC_ gene and other resistance genes, including *qnrs*, *tetA*, *tet34*, and *CTX*, were present in both strains. The resistance genes *KPC* and *qnrs* were found on distinct plasmids, mostly related to resistance to tetracycline, carbapenems, quinolones, and other antibiotics. Among the genes linked to virulence were *cirA*, *fimC*, and others. The primary virulence phenotypes included ferriferous carrier, transporter, lipopolysaccharide, fimbriae, and capsule. The genomic loop plots of plasmid 1 of the strain in this study were compared with those of virulence plasmid 1 of NUHL30457 (Fig. 6). However, there were no discernible differences. Thus, we performed genomic SNP analysis of the two strains to determine the potential reasons for virulence polymorphism, which demonstrated that *VirB11* was the differential gene between the two strains (Table S1).

### Proteomic analysis of CRKP13 and CRKP41

**Fig 7:**
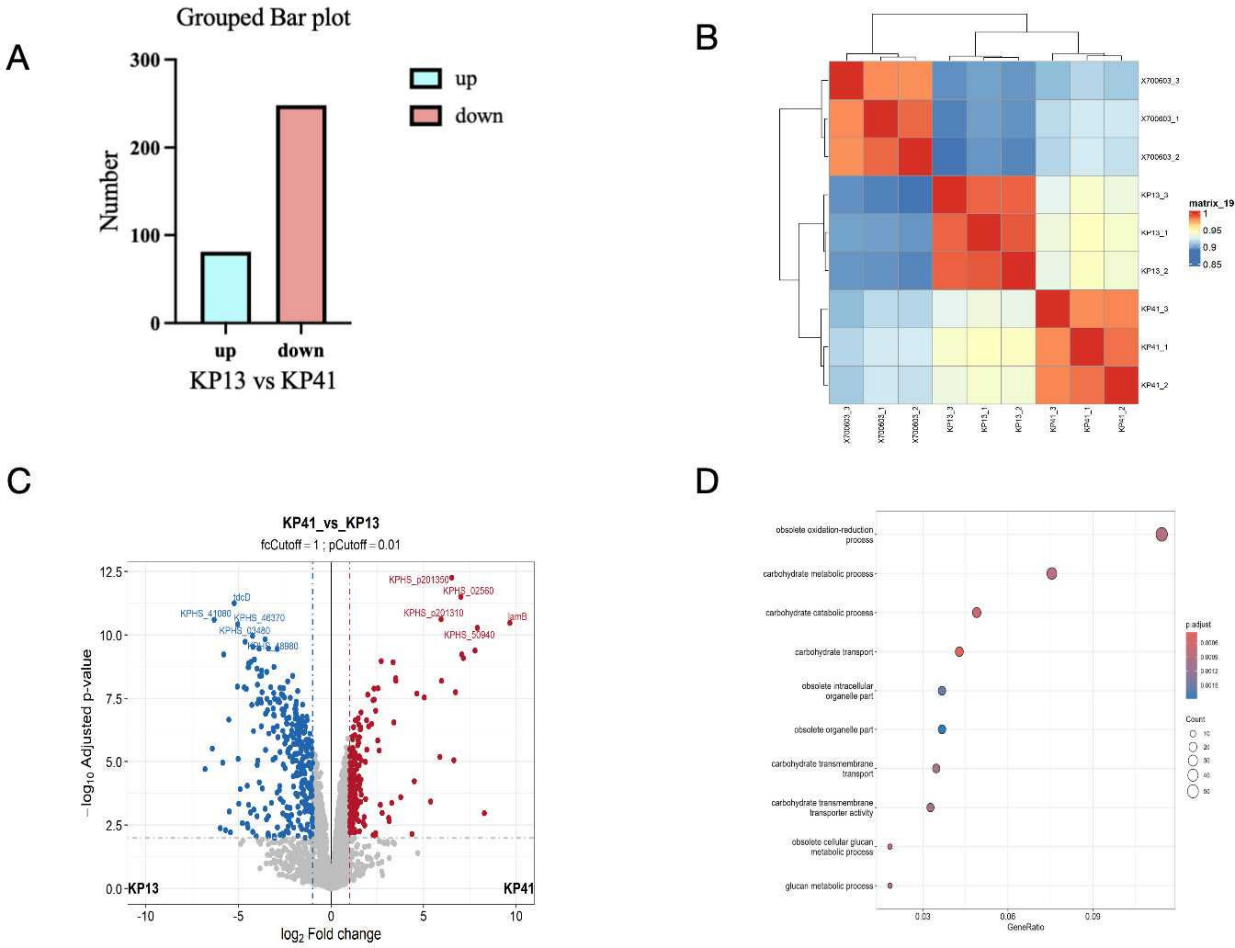
Overview of *Klebsiella pneumoniae* proteome data set. (**A**) The different comparison groups of the horizontal coordinate. The vertical coordinate is the number of significantly different proteins. Red represents the number of upregulated proteins, and light blue represents the number of downregulated proteins. (**B**) Correlation analysis of KP13 and KP41 protein samples. The color represents the Pearson correlation coefficient; the closer to red, the stronger the correlation, and the closer to blue, the weaker the correlation. It can be seen from the figure that KP13 has the least difference between groups, and KP41 has the most difference between groups. (**C**) Volcano plots of differentially expressed proteins (DEPs) between CRKP13 and CRKP41. Differential proteins were screened according to |log2 fold change| > 0.585 and p.adj < 0.05. The gray protein was not significantly different; the red protein was significantly upregulated; and the blue protein was significantly downregulated. (**D**) KEGG enrichment of DEPs retained by overlap analysis and validation of the selected genes.

The mass spectrometry procedure primarily encompassed protein extraction, peptide analysis, and LC-MS/MS using data acquisition and database retrieval in our setting. Peptide and liquid chromatography exhibited 3,268 proteins from the control and two strains (complex proteins). Significant changes were observed, with 81 proteins upregulated and 248 proteins downregulated (Fig. 7A). The correlation analysis of KP13 and KP41 protein samples revealed that KP13 exhibited the smallest intergroup variance, whereas KP41 demonstrated the greatest intergroup variance (Fig. 7B). The volcano map indicated notable disparities in proteins between the two groups (Fig. 7C). A significant number of related virulence proteins were identified compared to drug-resistant proteins between the two groups with cluster interaction proteins (Supplemental Table S2). The majority of the discovered differentially expressed proteins were involved in energy metabolism, cellular activities, catalytic activity, and binding. The Gene Ontology functional enrichment analysis of differentially expressed proteins using the Fisher exact test indicated distinct protein expression profiles. The highest concentration of differentially expressed proteins was linked to energy metabolism (Fig. 7D)

### Real-time quantitative PCR for transcription analysis and Sanger sequencing

**Fig 8:**
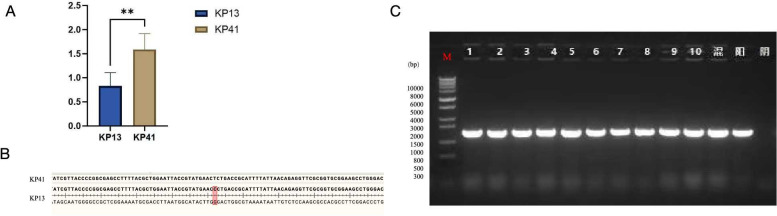
Real-time quantitative PCR for transcription analysis and Sanger sequencing. (A) Real-time quantitative PCR analysis of the VirB11 gene in the KP13 and KP41 strains. The expression of the *VirB11* gene in the two strains was statistically significant. (**B**) Base difference of the *VirB11* gene between the KP13 and KP41 strains. *VirB11* of the two strains showed that C was mutated to G at 712 base. (**C**) PCR gel electrophoresis detection of the *VirB11* gene in the KP13 strain (lane M: DNA molecular weight marker; lanes 1–10: strains to be tested; mixture: mixture of KP13 and mutant strains; yang: positive control; yin: negative control. **P < 0.01.

The SNP and indel results showed transcriptomic expression in the two strains of the *VirB11* gene. The qPCR results indicated a statistically significant difference in the *VirB11* gene between the two strains (Fig. 8A). The Sanger sequencing of *VirB11* in the two strains revealed that the KP13 strain was mutated from C to G at nucleotide 712 (Fig. 8B). In addition, we validated that the virulence polymorphism resulted from a single base mutation in the gene of the two strains.

### Research on the effects of *VirB11* gene mutations on virulence and growth

**Fig 9:**
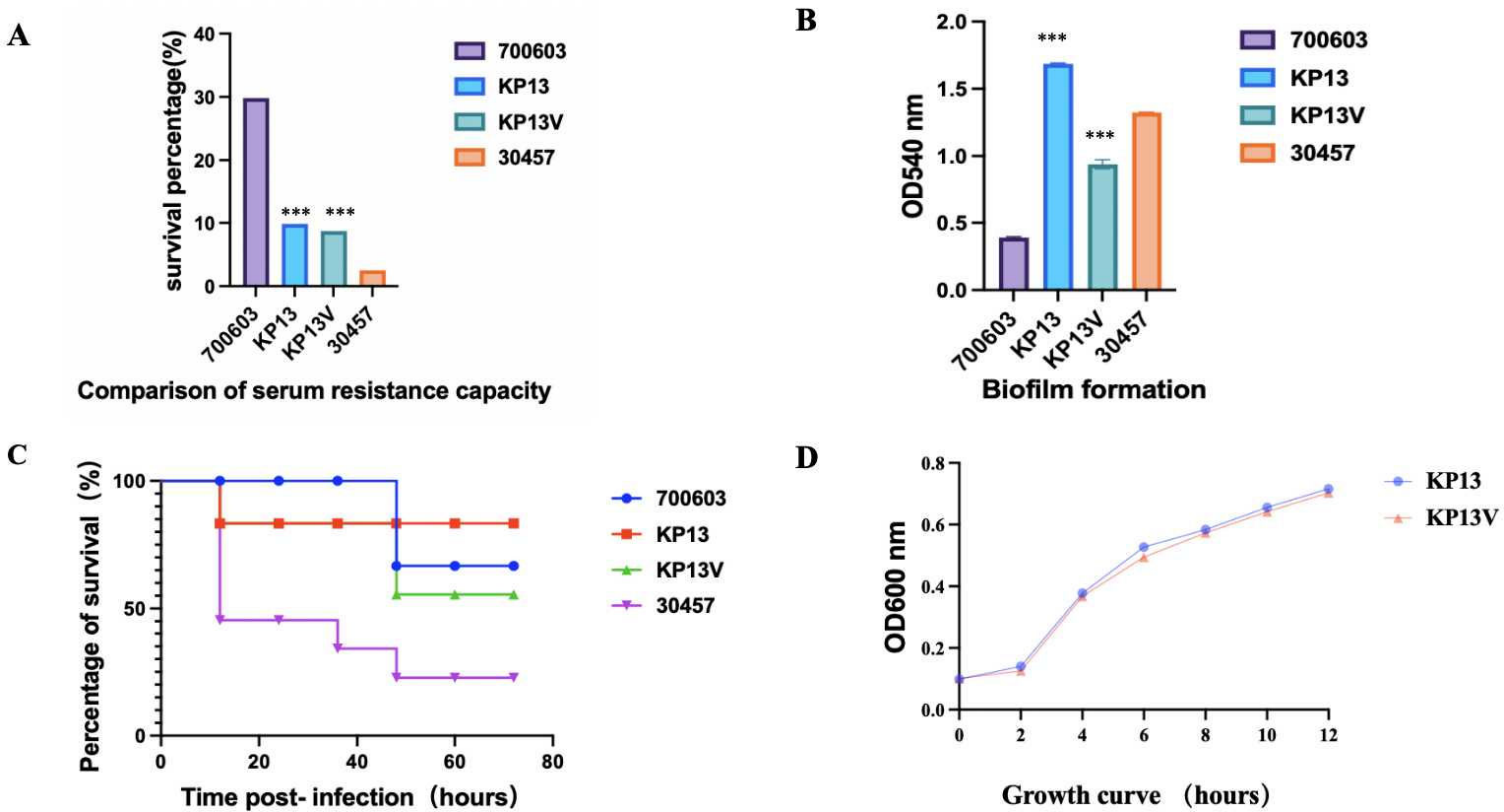
Virulence phenotypes of KP13 and KP13V strains. (A) Serum resistance capability of KP13 and KP13V. The serum resistance of KP13 is higher than that of KP13V. (**B**) The biofilm-forming ability of KP13 is higher than that of KP13V. (**C**) Virulence analysis of strains KP13 and KP13V in the *Galleria mellonella* infection model. (**D**) Growth ability of KP13 and KP13V. There was little difference in the growth ability of the two strains under the same conditions and concentration. ***P < 0.001.

In this study, CRISPR/Cas9 gene editing technology was used to successfully mutate the G base at site 712 in the KP13 strain to C base (see Table S2 for all primers). PCR identification can amplify 679 bp size gene fragments (Fig. 8C). Simultaneous sequencing results showed successful point mutations (supplemental materials). The survival rate of KP13V was higher than that of KP13, and the serum resistance and biofilm formation ability of KP13 were weaker than that of KP13, indicating that *VirB11* gene mutation of KP13 would affect the virulence of the strain, further proving that the VirB11 gene may be the cause of virulence polymorphism of the KP13 and KP41 strains from the same source. There was little difference in the growth ability of the two strains under the same conditions and concentration, indicating that the *VirB11* gene did not affect the growth characteristics of the strains (Fig. 9).

### Comparative analysis of the surrounding environment of the *KPC* gene

**Fig 10:**
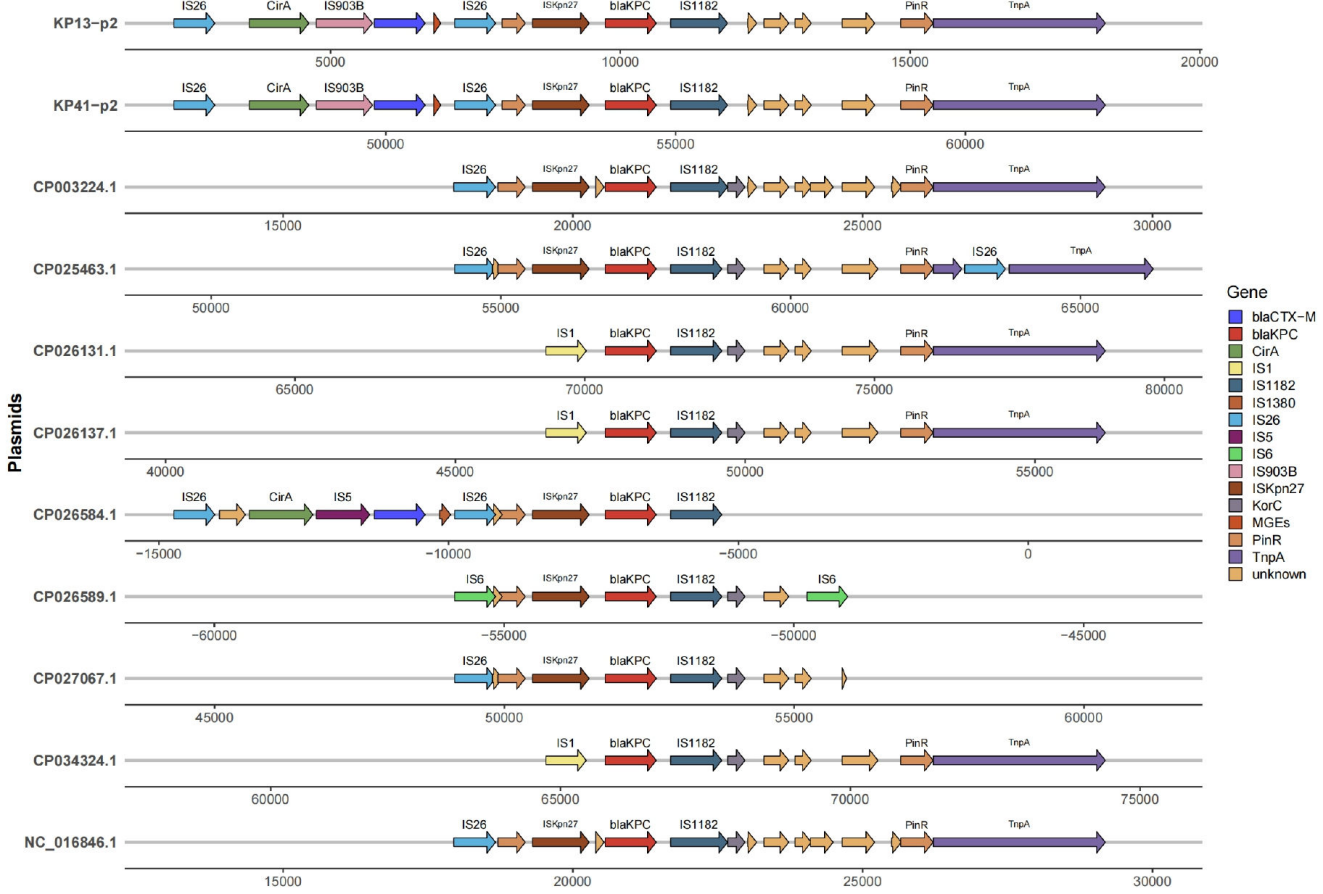
Surrounding environment of *KPC* gene comparison of pKPC sequences carried by strains KP13 and KP41. The vast majority of strains in the surrounding environment of the KPC gene have IS26 insertion elements upstream and IS1182 insertion elements downstream; only a few strains have IS1 insertion elements upstream and IS1182 insertion elements downstream.

The genetic environment of the *KPC* gene carried by strains KP13 and KP41 and other *Klebsiella pneumoniae* revealed that the two strains’ upstream and downstream elements were IS26, ISKpn27, and IS1182 (Fig. 10).

## DISCUSSION

*Klebsiella pneumoniae* is one of the major pathogens that causes pneumonia and is naturally present in the gut and respiratory tract of healthy individuals. *Klebsiella pneumoniae* can exhibit a highly resistant or virulent phenotype by acquiring drug-resistant or virulent plasmids (11, 17). Here, we analyze the CR-hvKP strain from the same source to understand the virulence polymorphism mechanism. We selected four groups based on the same source of PFGE, MLST, and K out of 54 strains. The mechanistic findings of our investigation revealed that the virulent phenotypes of these groups were polymorphic.

The absence of a highly virulent phenotype in specific CRKP isolates that possess virulent plasmids may be a consequence of mutations in the *rmpA* and *rmpA2* genes, which result in a loss of their original phenotypic function. This suggests that there may be additional factors that contribute to the high virulence of CRKP (5, 11). Research has demonstrated that the external environment, antibiotic selection pressure, or bacterial self-regulation can result in a series of modifications to bacteria’s viability, stability, and drug resistance (18). Our analysis of the results indicated that all four strains, which were from the same source group, had a poor prognosis, a death rate of 54.5%, longer hospital stays, a predominantly elderly infected population, a high prevalence of basic diseases and immune status changes, underlying diseases, and combined antibiotic use, suggesting consistency with prior research. Additionally, the formation and complications of resistance in β-lactam-producing bacteria, as well as the recombination of β-lactam resistance genes with other antibiotic resistance genes and virulence genes, were substantially increased by the long-term and substantial use of β-lactam antibiotics by all patients in our study. Thus, we posited that the virulent polymorphism of the strain may be associated with the patients’ baseline conditions and the surrounding environment, including the hospitalized department and antibiotic environment.

The lateral transfer of exogenous conjugative plasmids or transposons is linked to virulence polymorphisms in these strains (19). Thus, we subsequently categorized the immune status of our study patients into hyperactive, normal, and impaired groups based on their diseases and immunoglobulin, complement, and interleukin levels to correlate the potential difference between the strain’s virulent polymorphism and immune status. The whole-gene sequencing results of our investigation indicated that strains KP13 and KP41 exhibited no difference in the type and quantity of drug resistance and virulence genes.

Next, the *KPC* resistance genes, strains harboring *qnrs*, *tetA*, and *TEM-1*, and virulent genes such as *fimA*, *entB*, *wcaG*, *peg-344*, *iucA*, *iroB*, and *iutA* were analyzed in our study patients. Among them, the resistance genes *KPC* and qnrs were located on different plasmids, suggesting that the strain contained a significant number of plasmids and virulent genes. These virulent genes accelerate bacterial virulence evolution for environmental adaptation and survival. Considering the clinical prognosis and antibiotic administration in our study patients, we postulated that the existence of drug-resistant or virulent plasmids in bacteria could serve as a potential vector for transmission to other bacteria and humans, thereby jeopardizing the efficacy of conventional antibiotics in managing clinical infections (14, 20). This stresses the necessity for infection control protocols to avert the proliferation of CRKP strains in healthcare settings.

The *KPC* gene’s genomic context includes IS26 and ISKpn27 elements upstream, as well as IS1182 elements downstream. The synergistic effect of carbapenemase and IS insertor increases the possibility of bacterial drug-resistant plasmid transfer to some extent and amplifies the resistance of the bacterial *KPC* gene (20, 21), complicating carbapenem resistance treatment and providing a better understanding of the diversity and evolution of this type of plasmid. Consequently, we identified 2,518 genes from the KP13 and KP41 strains using KEGG. Our findings demonstrated a robust link among the majority of annotated genes, basic life processes, bacterial signal transduction, bacterial proliferation and mortality, transport mechanisms, and catabolism. Nevertheless, despite the two strains exhibiting identical gene annotation functions and gene counts, a substantial disparity in pathogenicity was observed. KP13 and KP41, exhibiting markedly distinct virulences, were selected from the PFGE-ST11-K2 group for supplementary bioinformatics analysis to elucidate the potential factors contributing to virulence polymorphism across strains of the same origin. The outcomes of our study indicated that although the three plastids and one chromosome of the two strains were comparable in size, one plasmid exhibited a much bigger and distinct size, prompting us to investigate a potential correlation with the strains’ virulence polymorphism.

The gene sequences of plasmid 1 from strains NUHL30457 KP13 and KP41 were analyzed to ascertain its aggressiveness. Plasmid p1 from both strains exhibited identical sequences to strain NUHL30457 (GenBank: CP026587.1). The high homology and non-exclusivity do not elucidate the infectious nature of the strains. Whole-genome sequencing and comparative genomic research were conducted to verify the virulence polymorphism among the strains. No statistically significant difference in KEGG functional classifications was seen among the strains, indicating the absence of notable genome deletions or amplifications. Genomic studies of these two strains identified 11 SNP variants, mainly located within *PduC* and *VirB11*. Annotation studies showed that there were two nonsense mutations and nine non-synonymous modifications, as well as transcription factors, transport subunits, oxidizing kinases, and reductases. *VirB11* functions as an ATPase that modulates fimbriae and flagella. We enhanced proteomics and *VirB11* gene expression in both strains to validate the findings. The two strains had distinct *VirB11* genes, as determined by qPCR. Sanger sequencing of the *VirB11* gene in both strains identified a C-to-G mutation at nucleotide 712. The proteomic results indicated alterations in the expression of signaling pathways associated with energy metabolism among the strains. In this study, the VIrB11 gene of the KP13 strain was mutated from C to G at the 712 position, and it was found that the virulence of the KP13V strain after mutation was changed compared with that of KP13, but there was no significant change in growth. This further indicates that virulence gene virulence 11 is the possible cause of virulence polymorphism of two strains of the same origin in this study, but it does not exclude the external environmental influences, such as antibiotics where the strains are located. Future studies need to pay more attention to the external influencing factors of the strains of the same origin.

The study contains several flaws, including a small sample size, an excessive focus on a single-gene mutation, the absence of other potential factors, the inability to test the infectiousness of the mutation in humans or animals, and the lack of a comprehensive discussion or long-term observation of how gene mutations function. Consequently, to improve the comprehensiveness and accuracy of future research, it is essential to meticulously examine the dynamic mechanisms of bacterial virulence under diverse environmental situations utilizing accurate genomic and metabolic techniques.

In summary, environmental influences, gene mutations, horizontal gene transfer, epigenetic modifications, and other variables may result in variations in pathogenicity, despite the strains’ identical genetic background. Our research suggests that virulence variations between isolates of identical origin may be attributed to mutations in the *VirB11* gene. Variations in virulence can result from mutations in virulence genes, which can result in the production of novel virulence factors in bacteria or alter their responses to the host. Consequently, it is essential to analyze not only the strain itself but also the external environment, heterogeneous underlying conditions, varying immune statuses, and the specific therapeutic dosage and duration of host antimicrobial agents during clinical anti-infective treatment. Additionally, understanding the mechanism of the CR-hvKP virulence plasmid may provide a novel perspective and establish a fundamental foundation for the prevention and management of nosocomial infections.

## Data Availability

The sequences of the CRKP13 and CRKP41 strains are available in the NCBI database under accession numbers PRJNA1153628 and PRJNA1153625.

## SUPPLEMENTAL MATERIAL

Table S2 legend spectrum.02464-24-s0001.docx Description of Table S2.

Table S1 spectrum.02464-24-s0002.xlsx SNP analysis of KP13 and KP41.

Graphical abstract spectrum.02464-24-s0003.pdf Visual diagram of study.

Table S1 legend spectrum.02464-24-s0004.docx Description of Table S1.

Table S2 spectrum.02464-24-s0005.xlsx KP13- and KP41-related differential protein expression and gene annotation.

ASM does not own the copyrights to Supplemental Material that may be linked to, or accessed through, an article. The authors have granted ASM a non-exclusive, world-wide license to publish the Supplemental Material files. Please contact the corresponding author directly for reuse.
