# Supplementary material for: Mechanism of virulence polymorphism in CR-hvKP strains from the same source
Source: Microbiol Spectr. 2025 May 23;13(7):e02464-24. doi: 10.1128/spectrum.02464-24 (PMC12210850; doi:10.1128/spectrum.02464-24)
Supplement: Table S2 legend — Description of Table S2. [file spectrum.02464-24-s0001.docx]

Enrichment indicates the proportion of all differential genes of the two strains among all background genes or substances; and functional annotation of each differential gene in the KEGG database
