# Supplementary figures and images for: Mechanism of virulence polymorphism in CR-hvKP strains from the same source
Source: Microbiol Spectr. 2025 May 23;13(7):e02464-24. doi: 10.1128/spectrum.02464-24 (PMC12210850; doi:10.1128/spectrum.02464-24)

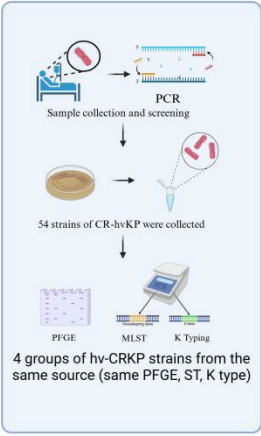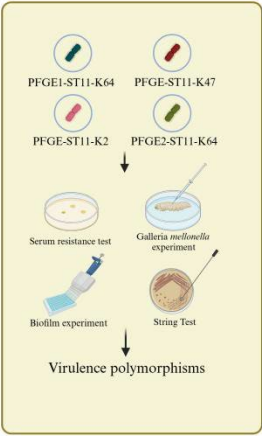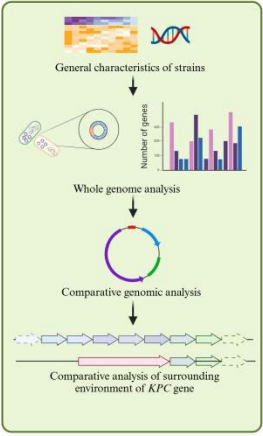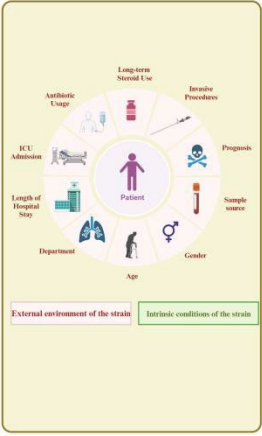

Supplement: Graphical abstract — Visual diagram of study. [file spectrum.02464-24-s0003.pdf]
